# Prevention and control of *Aedes* transmitted infections in the post-pandemic scenario of COVID-19: challenges and opportunities for the region of the Americas

**DOI:** 10.1590/0074-02760200284

**Published:** 2020-08-05

**Authors:** Héctor Gómez Dantés, Pablo Manrique-Saide, Gonzalo Vazquez-Prokopec, Fabian Correa Morales, João Bosco Siqueira, Fabiano Pimenta, Giovanini Coelho, Haroldo Bezerra

**Affiliations:** 1 National Institute of Public Health, Cuernavaca, Mexico; 2 Universidad Autónoma de Yucatan, Yucatan, Mexico; 3 Emory University, Department of Environmental Sciences, Atlanta, GA, United States of America; 4 Ministry of Health, Mexico; 5 Universidade Federal de Goiás, Goiânia, GO, Brasil; 6 Secretaria de Saúde de Belo Horizonte, Belo Horizonte, MG, Brasil; 7 Pan-American Health Organization/World Health Organization, Department of Communicable Diseases and Environmental Determinants of Health, Neglected, Tropical and Vector-Borne Diseases, Washington, United States of America

**Keywords:** COVID-19, dengue, Zika, Chikungunya

## Abstract

The coronavirus disease of 2019 (COVID-19) pandemic challenges public health systems around the world. Tropical countries will face complex epidemiological scenarios involving the simultaneous transmission of severe acute respiratory syndrome coronavirus 2 (SARS-CoV-2) with viruses transmitted by *Aedes aegypti*. The occurrence of arboviral diseases with COVID-19 in the Latin America and the Caribbean (LAC) region presents challenges and opportunities for strengthening health services, surveillance and control programs. Financing of training, equipment and reconversion of hospital spaces will have a negative effect on already the limited resource directed to the health sector. The strengthening of the diagnostic infrastructure reappears as an opportunity for the national reference laboratories. Sharing of epidemiological information for the modeling of epidemiological scenarios allows collaboration between health, academic and scientific institutions. The fear of contagion by COVID-19 is constraining people with arboviral diseases to search for care which can lead to an increase in serious cases and could disrupt the operation of vector-control programs due to the reluctance of residents to open their doors to health personnel. Promoting intense community participation along with the incorporation of long lasting innovations in vector control offers new opportunities for control. The COVID-19 pandemic offers challenges and opportunities that must provoke positive behavioral changes and encourage more permanent self-care actions.

Since the identification of the new coronavirus (severe acute respiratory syndrome coronavirus 2 - SARS-CoV-2) and associated disease (coronavirus disease of 2019 - COVID-19) in January 2020 in China, the virus has spread to all continents and the World Health Organization (WHO) has reported more than 3.2 million infections and 233.6 thousand deaths (as of May 1).^1^ ^,^ ^2^ Unprecedented measures to contain the pandemic and the response of the health systems are being implemented by the countries with a primary focus at the national level. At the local level, hand washing, respiratory hygiene recommendations, and physical distancing are key practices for disease containment, mitigation and suppression. The closing of the borders and social events in urban spaces have also contributed to mitigate the transmission,^3^ although its implementation has been very heterogeneous in each of the affected countries. There is discrepancy between predictive models of COVID-19 behavior; some estimate a significant decrease in transmission between June and September, and on the contrary, additional transmission waves are also forecasted for the second semester of 2020.^4^ ^,^ ^5^ The high occurrence of COVID-19 in tropical cities of Latin America^6^ reflects the heterogeneous response of political leaders and the national capacity of public health systems in those countries. The transitions will thus vary accordingly from country to country.^7^

The unexpected expenditures disbursed to implement the prevention and control measures of COVID-19 has put countries in a crisis that will affect not only economic activities but also the regular financing of routine preventive and control programs. We identify that this will be an additional challenge for the health systems and the economy of the countries of Latin America and the Caribbean (LAC). Contrary to what will happen in temperate countries, a large population contingent from tropical countries will experience complex epidemiological scenarios that will involve the simultaneous or intercalated transmission of SARS-CoV-2 with viruses of dengue, Zika and Chikungunya transmitted by *Aedes aegypti* (ATDs).^8^ ^,^ ^9^

Another notable aspect is the potential exhaustion of the surveillance systems to investigate and report arboviral cases in a timely manner. In March 2020, the Pan American Health Organization (PAHO) issued an alert calling attention to the trend of increasing dengue cases in at least 27 countries of the Region compared to 2019.^10^ Dengue data obtained from Health Information Platform for the Americas (PLISA) show that weekly dengue cases during 2020 were reported on a higher rate than the weekly average of dengue cases reported during the 2014-2019 period, and a sharp decrease in notification of confirmed dengue cases started along with the report of COVID-19 cases in the LAC Region^11^ (Figure). The decrease in notification was particularly remarkable for South and Central America.

**Figure:**
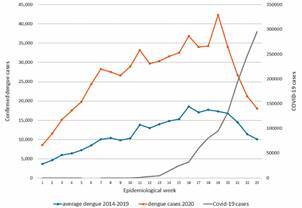
Number of confirmed case of dengue and COVID-19 reported in the Americas by week (2018-2020). Source: Pan American Health Organizations. Consulted data from May 13, 2020 on https://www.paho.org/es.

Despite having limited information on the direct impact on human health of the interaction of arboviral diseases with COVID-19,^12^ it is essential that all efforts be made to protect populations at risk^13^ since *Aedes-*transmitted diseases (ATDs) mainly affect vulnerable populations living in poor urban or rural areas and in houses with limited access to sewerage and drinking water services.^14^ ^,^ ^15^ The indirect impact of COVID-19 on arboviral diseases may be even greater than the direct one.

Health seeking behavior has been dramatically modified by COVID-19, driven by fear of contagion in the population, but also by messages from the health care authorities who recommend staying at home until severe symptoms (breathing problems) develop. In contrast, dengue cases are encouraged to present to health care units for close and early clinical monitoring (i.e., daily hematocrit). Clinical management and rapid diagnosis of both diseases in the context of appropriate health facility triage and case management must be developed. For example, maintaining separate care facilities when possible for COVID-19 and arboviral diseases is highly recommended.

On the other hand, the fear of COVID-19 contagion could also disrupt the operation of vector-control programs. Residents may be reluctant to open their doors to health personnel and health brigades may not want to visit SARS-CoV-2 high risk areas because of lack of personal protection equipment and potential exposure to the virus. The evaluation of peridomestic settings arises as an alternative by vector control managers but avoiding the interaction with the community.

*Challenges and opportunities* - The simultaneous occurrence of *Aedes-*transmitted diseases and COVID-19 in the LAC region presents us with very important challenges but also offers opportunities for strengthening health services, surveillance and control of epidemics (Table).

**TABLE:** Comparative features of COVID-19 and arboviruses transmitted by *Aedes aegypti*

Clinical and epidemiologic conditions	COVID-19	*Aedes-*transmitted diseases (DEN, CHIK, ZIK ,YF)
Case management		
Case detection	Asymptomatic and clinical	Symptomatic (febrile syndrome)
Asymptomatic infection	80% Very important in transmission	Variable very important in transmission
Mild	10%	20-40%
Moderate	5%	< 10%
Severe	5% Mandatory intensive therapy	5% intensive care
Lethality	2 to 5%	1-2%
Reproduction rate (Ro)	2-4	2-3
Health services burden	Hospital level	Primary health and hospital care
Public health measures		
Warning signs	fever, fatigue, coughing, pneumonia, difficulty breathing, acute respiratory distress	Alarma signs: abdominal pain or tenderness, persistent vomiting, clinical fluid accumulation, mucosal bleed, lethargy or restlessness, liver enlargement (> 2 cm)
Preventive measures	Hand washing, respiratory hygiene, face masks, personal protective equipment in health units	Elimination of unnecessary containers where standing water can accumulate Protection of water storage containers (lid, cleaning), use of personal protection methods (topical repellents, spatial repelents), house improvement (mosquito screens on doors and windows, tank covers etc)
Physical distancing	Indispensable	Not relevant
Isolation (quarantine)	Compulsory in confirmed cases	Partial, severe cases
Mobility Search for contacts	Mandatory, mass confinement Test, track, treat and isolate	Tracking contacts and mobility surveys
Urban concentration	Very important (density)	Very important (density)
Water supply	Constant hand washing	Avoid breeding sites
Sanitation	Surfaces, hands, face	Breeding sites control (disposal and clean-up) use of insecticides (chemical and biological)
Diagnostic testings	Test (PCR) nasal swab confirmation, track, treat and isolate contacts	Viral isolation, RNA (PCR), NS1 (ELISA, rapid diagnositics), antibody: IgM/IgG usually ELISA or rapid diagnositc. Antigen tests relevant early Antibody tests late test severe cases not for tracking purposes
Vaccine	Not available, fast development	Yellow fever, slow development

CHIK: Chikungunya; DEN: dengue; YF: yellow fever; ZIK: Zika.

The main challenge or obstacle, without a doubt, will be the economic crisis of the countries along with the reorganization of health services disrupted by the health emergency that diverted attention to the diagnosis and care of patients with severe respiratory manifestations. The reorganization of health services and the conversion of hospital spaces to contain the burden of disease caused by COVID-19 has been detrimental to the care of other problems that are equally relevant and demanding in terms of the daily demand for specialized medical services. A similar experience occurred with reproductive, maternal, and child health services during the recent Zika epidemic in the Americas, and it may well happen again in the future with the resurgence of COVID-19 or any other epidemic of global proportions. Lessons learned from these experiences should help us prepare for future health contingencies. Particular attention should be put in sustainability of interventions along with gender issues regarding risk of transmission, health care options and control responsibilities in the domestic and urban settings.^16^

Financing of training, equipment and reconversion of hospital spaces will have a negative effect on already the limited resources directed to the health sector at the continental level. The indirect impact on health in general will be a collateral cost that will have to be evaluated in due course. There will be countries in the Americas that can face this challenge better than others but the countries of the region have been hit by other epidemics without managing to recover financially from recurring health crises: cholera, H1N1 influenza, Zika, Chikungunya or dengue.

For the epidemiological surveillance systems there are challenges and opportunities given the possible seasonal coincidence of transmission, since the triggering signs and symptoms of seeking medical attention in the COVID-19 charts, are fever, general malaise and respiratory discomfort also frequent in arboviruses. The high occurrence of COVID-19 in tropical cities (e.g., Iquitos, Peru) indicates that temperature may not be as limiting for transmission as some hypothesize, however, the differential diagnosis of dengue, Zika and Chikungunya will need to incorporate COVID-19 as a diagnostic possibility, although its confirmation triggers very different control actions. The strengthening of the diagnostic infrastructure reappears as an opportunity to prepare the capacity of the regional, nation and state reference laboratories to diagnose a very wide spectrum of infections agents for which the technical inputs, equipment or trained personnel are not available. Thus, interaction between academic and public health institutions is mandatory as the pandemic has demonstrated.

Similarly, proliferation and real-time virtual epidemiological information platforms developed to monitor the pandemic must be available to monitor arboviral diseases and other infectious diseases with the same intensity and frequency with which they have been occurring in mapping the progress of the COVID-19 pandemic. The effort to share and integrate epidemiological information sources for the construction and modeling of epidemiological scenarios becomes an imperative for regional health that allows collaboration between health entities with academic and scientific institutions. Examples of such synergy include timely situational analysis, the generation of adequate risk scenarios for the design and selection of more effective control interventions as well as the use of information for education and communication campaigns with the population. An area of opportunity would be the incorporation of communication technologies (TICs) in the test and tracing of contacts as well as mobility surveys in support of the early warning surveillance systems.

Traditionally, the countries of the American region based their control activities with the involvement of the community for the removal and control of domestic larval habitats The development of protective kits or tools so family members can perform the application of insecticide by themselves (spatial repellents, spray bombs) and the free distribution or purchase by the family of appropriate consumer products like spatial repellents, insecticide treated materials, or screens on doors and windows are interventions that should be encouraged and tested on a wider scale. Given the new circumstances, home visits could be interrupted by physical distancing and it is essential to start incorporating new control strategies that do not depend so much on home visits by health personnel and are better supported by the promotion of domiciliary interventions that may be developed inside the house by the family nucleus. An additional opportunity is the more efficient use of resources that is based on risk stratification targeting areas in urban settings that are responsible for more than 50% of historical cases.^17^

In the case of *Aedes aegypti* surveillance and control, the possibility is opened for promoting the incorporation of innovations that do not require and/or can reduce the constant presence of health personnel.^18^ For vector surveillance, the use of rapid larval/pupal surveys or the use of ovitraps in the peridomestic environment with the support of the community could be implemented to improve monitoring of vector densities while the proactive use of targeted indoor residual spraying (TIRS)^19^ and contact tracing,^20^ for example, would favor a more extensive and long-lasting control. This paradigm shift becomes imperative and countries in the region have already drawn up action plans for the concurrence of COVID-19 and ATDs.^21^ ^,^ ^22^

Of course, the nature of ATDs will also require action in response to outbreaks. The responsive actions are well-known in each country and must be carried out in a timely manner and have the human, consumables, and financial resources for it. Recommendations established in the operational guides must be followed. Chemical control (larvicides and adulticides) should be properly applied and selected based on evidence of susceptibility of the local vector populations to guarantee its effectiveness.

In the field of risk communication, the COVID-19 pandemic leaves us with lessons, challenges and opportunities that must lead to better information campaigns. Risk communication strategies that increase positive behavioral changes that combat disinformation and encourage people to incorporate permanent self-care actions and not only in crisis situations. At the same time, the risks of transmission of COVID-19 and its dispersion throughout the territory limit the full development of the activities that require the action of health agents, but it also creates a great opportunity to promote effective participation of population with the incorporation of protective habits of prevention and maintenance of the domestic environment free of risk factors for the presence of vectors. Vector control personnel must be considered essential, and their activities must continue to support the actions required for the effective prevention and control of VTDs, even within the contingency imposed by COVID-19.

Given the possible scenario of simultaneous transmission of the ATDs and COVID-19 agents, it is important that the planning of the actions be integrated, combined with the effective multisectoral and population participation where the public and private sectors, schools, the media, tune into the common strategy to deal with health problems. Recently, the countries of the Region of the Americas, with the support of the Pan American Health Organization (PAHO), unanimously approved the Plan of Action on Entomology and Vector Control 2018-2023,^23^ whose objective is to strengthen regional and national capacity for prevention and control of key vectors, and reduce the spread of vector borne diseases (VBD). This Plan of Action is based on solid technical foundations that seek to strengthen inter-programmatic action, mobilize governments and communities, the improvement of entomological surveillance, the incorporation of new technologies, and the training, creation, and expansion the workforce. Given the emergence of COVID-19, it is essential that countries commit to supporting this Plan of Action and strengthen their surveillance and control programs in an integrated way where new epidemiological circumstances are seen as challenges and opportunities and much less as disbeliefs or limitations. Opportunities to improve control of ATDs will depend on how well we respond to the COVID-19 crisis and even though the pandemic is still in progress, the challenges ahead demand resources and new capabilities not only to cope with COVID-9 but to strengthen the overall capacity of the health system to respond to any new sanitary crisis.
